# The prevalence of premenstrual dysphoric disorder in patients with depressive or panic disorder

**DOI:** 10.1192/j.eurpsy.2022.1162

**Published:** 2022-09-01

**Authors:** Y. Chochev, R. Iakimova, M. Pandova

**Affiliations:** 1 UMHATNP St. Naum, Second Psychiatric Clinic, Sofia, Bulgaria; 2 Medical University Sofia, Faculty Of Medicine, Department Of Psychiatry And Medical Psychology, Sofia, Bulgaria

**Keywords:** PMDD, depressive disorder, panic disorder, comorbidity

## Abstract

**Introduction:**

Premenstrual dysphoric disorder (PMDD), a severe form of premenstrual syndrome (PMS), affects 3-5% of the women of childbearing age. According to scientific literature, the prevalence of PMDD increases with age and among the psychiatric patient population as well, e.g. in women suffering depressive disorder (DD) or panic disorder (PD).

**Objectives:**

To estimate the prevalence of PMDD in women without psychiatric comorbidities and those with concomitant DD or PD.

**Methods:**

A cross-sectional non-interventional study that enrolled 159 women, divided in 3 groups: 1) 98 women (mean age 31.04 ± 6.31) with PMS and no psychiatric comorbidities; 2) 31 women with PMS and DD (mean age 39.4±7.21); 3) 30 women with PMS and PD (mean age 31.2±7.89). PMS was assessed by the PSST (Premenstrual Symptoms Screening Tool). DD and PD were diagnosed by MINI and a psychiatric evaluation. Descriptive and frequency statistics were performed.

**Results:**

Within the group without comorbidities mild PMS was present in 48% (N=47) of the cases, moderate - in 41,8% (N=41), and in 10,2% (N=10) of the cases PMDD was diagnosed. Within the group with comorbid DD 25,8% (N=8) had mild PMS, 58,1% (N=18) had moderate and 16,1% (N=5) had PMDD. Among the women with comorbid PD 56,7% (N=17) suffered moderate PMS, 43,3% (N=13) - PMDD and no mild cases were documented.

**Conclusions:**

The results demonstrate that comorbid DD or PD increases the prevalence of PMDD. It is considerably more common in patients with PD than those with DD.

**Disclosure:**

No significant relationships.

